# Supplementary material for: Evaluation of molecular characterization and phylogeny for quantification of Acanthamoeba and Naegleria fowleri in various water sources, Turkey
Source: PLoS One. 2021 Aug 26;16(8):e0256659. doi: 10.1371/journal.pone.0256659 (PMC8389491; doi:10.1371/journal.pone.0256659)
Supplement: S1 Table — (DOCX) [file pone.0256659.s001.docx]

| **S1 Table.** Various water samples collected from İzmir region and their geographic coordination | | | | |
| --- | --- | --- | --- | --- |
| **Strain No.** | **Sample code** | **Sampling area** | **Locality** | **Geographic coordinates** |
| IWS1 | TW1 | Tap water | Menderes | 38.2564, 27.1352 |
| IWS2 | TW2 | Tap water | Bornova | 38.4572, 27.2104 |
| IWS3 | TW3 | Tap water | Torbalı | 38.2476, 27.5094 |
| IWS4 | TW4 | Tap water | Menderes | 38.0898, 27.2275 |
| IWS5 | TW5 | Tap water | Menderes | 38.0814, 27.1659 |
| IWS6 | TW6 | Tap water | Menderes | 38.0912, 27.1664 |
| IWS7 | TW7 | Tap water | Bornova | 38.4627, 27.2176 |
| IWS8 | TW8 | Tap water | Çeşme | 38.3546, 26.4598 |
| IWS9 | TW9 | Tap water | Çeşme | 38.3551, 26.4612 |
| IWS10 | TW10 | Tap water | Menderes | 38.1988, 27.1337 |
| IWS11 | TW11 | Tap water | Bornova | 38.4575, 27.2353 |
| IWS12 | TW12 | Tap water | Bornova | 38.4571, 27.2355 |
| IWS13 | TW13 | Tap water | Kemalpaşa | 38.4405, 27.3663 |
| IWS14 | TW14 | Tap water | Torbalı | 38.1624, 27.2921 |
| IWS15 | TW15 | Tap water | Torbalı | 38.1624, 27.2921 |
| IWS16 | TW16 | Tap water | Tire | 38.1646, 27.7460 |
| IWS17 | TW17 | Tap water | Buca | 38.3148, 27.2363 |
| IWS18 | TW18 | Tap water | Bornova | 38.4561, 27.2120 |
| IWS19 | TW19 | Tap water | Tire | 38.0889, 27.7332 |
| IWS20 | TW20 | Tap water | Kemalpaşa | 38.4338, 27.4042 |
| IWS21 | TW21 | Tap water | Karşıyaka | 38.5047, 27.1004 |
| IWS22 | TW22 | Tap water | Menderes | 38.2562, 27.1353 |
| IWS23 | TW23 | Tap water | Bornova | 38.4573, 27.2103 |
| IWS24 | TW24 | Tap water | Torbalı | 38.2477, 27.5095 |
| IWS25 | TW25 | Tap water | Menderes | 38.0897, 27.2276 |
| IWS26 | TW26 | Tap water | Menderes | 38.0813, 27.1658 |
| IWS27 | TW27 | Tap water | Menderes | 38.0911, 27.1662 |
| IWS28 | TW28 | Tap water | Bornova | 38.4626, 27.2175 |
| IWS29 | TW29 | Tap water | Çeşme | 38.3544, 26.4596 |
| IWS30 | TW30 | Tap water | Çeşme | 38.3552, 26.4610 |
| IWS31 | TW31 | Tap water | Menderes | 38.1985, 27.1335 |
| IWS32 | TW32 | Tap water | Bornova | 38.4574, 27.2355 |
| IWS33 | TW33 | Tap water | Bornova | 38.4569, 27.2354 |
| IWS34 | TW34 | Tap water | Kemalpaşa | 38.4404, 27.3660 |
| IWS35 | TW35 | Tap water | Torbalı | 38.1626, 27.2920 |
| IWS36 | TW36 | Tap water | Torbalı | 38.1623, 27.2922 |
| IWS37 | TW37 | Tap water | Tire | 38.1645, 27.7461 |
| IWS38 | TW38 | Tap water | Buca | 38.3149, 27.2360 |
| IWS39 | TW39 | Tap water | Bornova | 38.4563, 27.2123 |
| IWS40 | TW40 | Tap water | Tire | 38.0890, 27.7330 |
| IWS41 | TW41 | Tap water | Kemalpaşa | 38.4340, 27.4041 |
| IWS42 | TW42 | Tap water | Karşıyaka | 38.5048, 27.1003 |
| IWS43 | TW43 | Tap water | Seferihisar | 38.1951, 26.8422 |
| IWS44 | TW44 | Tap water | Selçuk | 37.9567, 27.3755 |
| IWS45 | PW1 | Pool water | Menderes | 38.2553,27.1360 |
| IWS46 | PW2 | Pool water | Menderes | 38.2553,27.1360 |
| IWS47 | PW3 | Pool water | Bornova | 38.4599, 27.2330 |
| IWS48 | PW4 | Pool water | Bayraklı | 38.4600, 27.1906 |
| IWS49 | PW5 | Pool water | Çeşme | 38.3512, 26.3009 |
| IWS50 | PW6 | Pool water | Konak | 38.4292, 27.1446 |
| IWS51 | PW7 | Pool water | Konak | 38.4280, 27.1452 |
| IWS52 | PW8 | Pool water | Konak | 38.4277, 27.1433 |
| IWS53 | PW9 | Pool water | Bornova | 38.4572, 27.2334 |
| IWS54 | PW10 | Pool water | Bornova | 38.4659, 27.2086 |
| IWS55 | PW11 | Pool water | Balçova | 38.3952, 27.0401 |
| IWS56 | PW12 | Pool water | Bornova | 38.4659, 27.2087 |
| IWS57 | PW13 | Pool water | Çiğli | 38.5135, 27.0390 |
| IWS58 | PW14 | Pool water | Menderes | 38.2554,27.1361 |
| IWS59 | PW15 | Pool water | Menderes | 38.2553,27.1360 |
| IWS60 | PW16 | Pool water | Bornova | 38.4600, 27.2330 |
| IWS61 | PW17 | Pool water | Bayraklı | 38.4601, 27.1907 |
| IWS62 | PW18 | Pool water | Çeşme | 38.3494, 26.3011 |
| IWS63 | PW19 | Pool water | Konak | 38.4294, 27.1445 |
| IWS64 | PW20 | Pool water | Konak | 38.4281, 27.1453 |
| IWS65 | PW21 | Pool water | Konak | 38.4272, 27.1430 |
| IWS66 | PW22 | Pool water | Bornova | 38.4570, 27.2332 |
| IWS67 | PW23 | Pool water | Bornova | 38.4660, 27.2085 |
| IWS68 | PW24 | Pool water | Balçova | 38.3953, 27.0400 |
| IWS69 | PW25 | Pool water | Bornova | 38.4660, 27.2088 |
| IWS70 | PW26 | Pool water | Çiğli | 38.5133, 27.0391 |
| IWS71 | WW1 | Well water | Menderes | 38.1532, 27.1801 |
| IWS72 | WW2 | Well water | Menderes | 38.1532, 27.1801 |
| IWS73 | WW3 | Well water | Bornova | 38.4623, 27.2136 |
| IWS74 | WW4 | Well water | Bornova | 38.4627, 27.2175 |
| IWS75 | WW5 | Well water | Buca | 38.3830, 27.1815 |
| IWS76 | WW6 | Well water | Buca | 38.3833, 27.1799 |
| IWS77 | WW7 | Well water | Kemalpaşa | 38.4408, 27.3688 |
| IWS78 | WW8 | Well water | Bayraklı | 38.4712, 27.1891 |
| IWS79 | WW9 | Well water | Torbalı | 38.2429, 27.3207 |
| IWS80 | WW10 | Well water | Dikili | 39.1091, 26.8726 |
| IWS81 | WW11 | Well water | Aliağa | 38.8649, 27.0598 |
| IWS82 | WW12 | Well water | Aliağa | 38.8649, 27.0598 |
| IWS83 | WW13 | Well water | Aliağa | 38.8660, 27.0586 |
| IWS84 | WW14 | Well water | Menderes | 38.2566, 27.1337 |
| IWS85 | WW15 | Well water | Karşıyaka | 38.4903, 27.0902 |
| IWS86 | WW16 | Well water | Menderes | 38.1533, 27.1804 |
| IWS87 | WW17 | Well water | Menderes | 38.1533, 27.1801 |
| IWS88 | WW18 | Well water | Bornova | 38.4624, 27.2135 |
| IWS89 | WW19 | Well water | Bornova | 38.4628, 27.2174 |
| IWS90 | WW20 | Well water | Buca | 38.3829, 27.1816 |
| IWS91 | WW21 | Well water | Buca | 38.3830, 27.1800 |
| IWS92 | WW22 | Well water | Kemalpaşa | 38.4403, 27.3690 |
| IWS93 | WW23 | Well water | Bayraklı | 38.4710, 27.1890 |
| IWS94 | WW24 | Well water | Torbalı | 38.2430, 27.3204 |
| IWS95 | WW25 | Well water | Dikili | 39.1089, 26.8724 |
| IWS96 | WW26 | Well water | Aliağa | 38.8650, 27.0599 |
| IWS97 | WW27 | Well water | Aliağa | 38.8649, 27.0598 |
| IWS98 | WW28 | Well water | Aliağa | 38.8659, 27.0585 |
| IWS99 | WW29 | Well water | Menderes | 38.2565, 27.1338 |
| IWS100 | WW30 | Well water | Karşıyaka | 38.4905, 27.0900 |
| IWS101 | WW31 | Well water | Menderes | 38.2565, 27.1337 |
| IWS102 | LW1 | Lake water | Menderes | 38.0776, 27.1624 |
| IWS103 | LW2 | Lake water | Menderes | 38.0770, 27.1669 |
| IWS104 | LW3 | Lake water | Konak | 38.4279, 27.1459 |
| IWS105 | LW4 | Lake water | Ödemiş | 38.3159, 28.0247 |
| IWS106 | LW5 | Lake water | Menderes | 38.0794, 27.1637 |
| IWS107 | LW6 | Lake water | Menderes | 38.0792, 27.1638 |
| IWS108 | LW7 | Lake water | Menderes | 38.0804, 27.1642 |
| IWS109 | LW8 | Lake water | Buca | 38.3472, 27.2411 |
| IWS110 | LW9 | Lake water | Buca | 38.3469, 27.2422 |
| IWS111 | LW10 | Lake water | Menderes | 38.0777, 27.1625 |
| IWS112 | LW11 | Lake water | Menderes | 38.0771, 27.1670 |
| IWS113 | LW12 | Lake water | Konak | 38.4281, 27.1460 |
| IWS114 | LW13 | Lake water | Ödemiş | 38.3160, 28.0250 |
| IWS115 | LW14 | Lake water | Menderes | 38.0795, 27.1638 |
| IWS116 | LW15 | Lake water | Menderes | 38.0793, 27.1640 |
| IWS117 | LW16 | Lake water | Menderes | 38.0800, 27.1644 |
| IWS118 | LW17 | Lake water | Buca | 38.3470, 27.2410 |
| IWS119 | LW18 | Lake water | Buca | 38.3468, 27.2424 |
| IWS120 | DW1 | Dam water | Ödemiş | 38.0842, 28.0678 |
| IWS121 | DW2 | Dam water | Menderes | 38.1732, 27.1606 |
| IWS122 | DW3 | Dam water | Menderes | 38.1606, 27.1501 |
| IWS123 | DW4 | Dam water | Menderes | 38.1597, 27.1509 |
| IWS124 | DW5 | Dam water | Ödemiş | 38.0845, 28.0676 |
| IWS125 | DW6 | Dam water | Menderes | 38.1730, 27.1600 |
| IWS126 | DW7 | Dam water | Menderes | 38.1603, 27.1500 |
| IWS127 | DW8 | Dam water | Menderes | 38.1599, 27.1510 |
| IWS128 | DW9 | Dam water | Seferihisar | 38.1791, 26.9086 |
| IWS129 | DW10 | Dam water | Seferihisar | 38.2200, 26.8799 |
| IWS130 | StW1 | Stream water | Menderes | 38.1402, 27.1966 |
| IWS131 | StW2 | Stream water | Menderes | 38.1402, 27.1966 |
| IWS132 | StW3 | Stream water | Menderes | 38.1402, 27.1966 |
| IWS133 | StW4 | Stream water | Torbalı | 38.1735,27.3800 |
| IWS134 | StW5 | Stream water | Menderes | 38.1400, 27.1963 |
| IWS135 | StW6 | Stream water | Menderes | 38.1401, 27.1965 |
| IWS136 | StW7 | Stream water | Menderes | 38.1403, 27.1964 |
| IWS137 | StW8 | Stream water | Torbalı | 38.1733,27.3801 |
| IWS138 | StW9 | Stream water | Selçuk | 37.9621, 27.2669 |
| IWS139 | SeW1 | Seawater | Selçuk | 37.9758, 27.2535 |
| IWS140 | SeW2 | Seawater | Urla | 38.2059, 26.6867 |
| IWS141 | SeW3 | Seawater | Karaburun | 38.5168, 26.6257 |
| IWS142 | SeW4 | Seawater | Çeşme | 38.3255, 26.3277 |
| IWS143 | SeW5 | Seawater | Selçuk | 37.9754, 27.2533 |
| IWS144 | SeW6 | Seawater | Urla | 38.2058, 26.6868 |
| IWS145 | SeW7 | Seawater | Karaburun | 38.5169, 26.6258 |
| IWS146 | SeW8 | Seawater | Çeşme | 38.3082, 26.3729 |
| IWS147 | TsW1 | Thermal spring water | Seferihisar | 38.0899, 26.9176 |
| IWS148 | TsW2 | Thermal spring water | Seferihisar | 38.0899, 26.9176 |
| IWS: Izmir water sample, TW: Tap water, PW: Pool water, WW: Well water, LW: Lake water, DW: Dam water, StW: Stream water, SeW: Seawater, TaW: Thermal spring water | | | | |
|  | | | | |
|  |  |  |  |  |
