# Supplementary material for: Evaluation of molecular characterization and phylogeny for quantification of Acanthamoeba and Naegleria fowleri in various water sources, Turkey
Source: PLoS One. 2021 Aug 26;16(8):e0256659. doi: 10.1371/journal.pone.0256659 (PMC8389491; doi:10.1371/journal.pone.0256659)
Supplement: S2 Table — (DOCX) [file pone.0256659.s002.docx]

| **S2 Table**. The set of primers and probes in 18S rRNA and ITS gene amplification for *Acanthamoeba* spp., *B.* *mandrillaris* and *N. fowleri* | | | | | |
| --- | --- | --- | --- | --- | --- |
| **Free-living amoeba** | **Gene** | **Primers and Probes** | **Sequence** | **Products** | **References** |
|  |  | AcantF900 | 5'-CCCAGATCGTTTACCGTGAA-3' |  | Qvarnstrom *et al*. (2006) |
| *Acanthamoeba* spp. | 18S rRNA | AcantR1100 | 5'-TAAATATTAATGCCCCCAACTATCC-3' | 180 bp |  |
|  |  | AcantP1000 | 5'-Cy5-CTGCCACCGAATACATTAGCATGG-BHQ3-3' |  |  |
|  |  |  |  |  |  |
| *Acanthamoeba* spp. | 18S rRNA | JDP1 | 5'-GGCCCAGATCGTTTACCGTGAA-3' | ∼500 bp | Schroeder *et al*. (2001) |
|  |  | JDP2 | 5'-TCTCACAAGCTGCTAGGGAGTCA-3' |  |  |
|  |  |  |  |  |  |
|  |  | BalaF1451 | 5'-TAACCTGCTAAATAGTCATGCCAAT-3' |  | Qvarnstrom *et al*. (2006) |
| *B. mandrillaris* | 18S rRNA | BalaR1621 | 5'-CAA ACT TCC CTC GGC TAA TCA-3' | 171-bp |  |
|  |  | BalaP1582 | 5'-FAM-AGTACTTCTACCAATCCAACCGCCA-BHQ1-3' |  |  |
|  |  |  |  |  |  |
|  |  | JBVF | 5'-AGGTACTTACGTTAGAGTGCTAGT-3' |  | Mull *et al.* (2013) |
| *N. fowleri* | 5.8S rRNA and ITS region (ITS1-ITS2) | JBVR | 5'-ATGGGACAATCCGGTTTTCTCA-3' | 123-bp |  |
|  |  | JBVP | 5'-FAM-ACGCCCTAGCTGGTTATGCCGGATT-BHQ1-3' |  |  |
|  |  |  |  |  |  |
| *Naegleria* spp*.* | ITS2 region | FW2 | 5′-GAACCT GCGTAGGGATCATTT-3′ | ∼450 bp | Pelandakis *et al*. (2000) |
|  |  | RV2 | 5′-TTTCTTTTCCTCCCCTTATTA-3′ |  |  |
|  |  |  |  |  |  |
