# Supplementary material for: Evaluation of molecular characterization and phylogeny for quantification of Acanthamoeba and Naegleria fowleri in various water sources, Turkey
Source: PLoS One. 2021 Aug 26;16(8):e0256659. doi: 10.1371/journal.pone.0256659 (PMC8389491; doi:10.1371/journal.pone.0256659)
Supplement: Attachment — Submitted filename: Response to Reviewers-090821.docx [file pone.0256659.s004.docx]

**Authors’ Responses to the Review Comments**

**Editorial Office:** Please review your reference list to ensure that it is complete and correct. If you have cited papers that have been retracted, please include the rationale for doing so in the manuscript text, or remove these references and replace them with relevant current references. Any changes to the reference list should be mentioned in the rebuttal letter that accompanies your revised manuscript. If you need to cite a retracted article, indicate the article’s retracted status in the References list and also include a citation and full reference for the retraction notice.

**Response to Editor Office:** As Editorial Office suggested, all references have been reviewed. Some of the references from the retracted articles were removed as follows and replaced with relevant current references.

" Kolören Z, Taş B. Molecular Characterization of Acanthamoeba species in Water Resources of Ordu Province in Turkey 2017" which is a reference in the reference list [27] has been removed.

Erdogan D.D., et al., 2020, unpublished reference has been replaced with relevant current references [52 and 53]. “[52] Ertabaklar H, Turk M, Dayanir V, Ertug S, Walochnik J. Acanthamoeba keratitis due to Acanthamoeba genotype T4 in a non-contact-lens wearer in Turkey. Parasitology research. 2007;100(2):241-6. doi: 10.1007/s00436-006-0274-0. PubMed PMID: 17013653.”

“[53].	Ozkoc S, Tuncay S, Delibas SB, Akisu C, Ozbek Z, Durak I, et al. Identification of Acanthamoeba genotype T4 and Paravahlkampfia sp. from two clinical samples. Journal of medical microbiology. 2008;57(Pt 3):392-6. doi: 10.1099/jmm.0.47650-0. PubMed PMID: 18287307.”

The following sentence and cite have been removed from the manuscript.

"Acanthamoeba T4 genotype was detected in the CSF sample of a patient with unknown cause of encephalitis in İzmir, Turkey (Aykur M., et. al., 2021, unpublished)"

“Also in previous study, we have reported the presence of N. fowleri genotype 2 from one patient in a cerebrospinal fluid sample (Aykur M., et. al., unpublished).”

**Additional Editor Comments:**

**Editorial Office:** In my opinion the article has been improved and deserves to be published pending few suggestions

**Response to Editor Office:** Many thanks for your time and interest in handling our manuscript. Many thanks for your insightful comments concerning our manuscript. They really helped our manuscript appear stronger. Specifically, we have revised our manuscript according to all your comments.

**Editorial Office:** Line 36: change “which” in “with”

**Response to Editor Office:** As Editorial Office suggested, line 36 “which” was changed as “with”.

**Editorial Office:** Lines 36-37: check the sentence; indeed the qPCR usually works on small fragments, the region that includes both 5.8S and ITS1-ITS2 could be a large fragment; so specify exactly which target gene/regions the primers are targeting.

**Response to Editor Office:** As Editorial Office suggested, qPCR specific primers target the ITS1 region and It has been corrected as follows. “*N. fowleri* positives were detected in six different water sources using qPCR with ITS regions (ITS1) specific primers”.

**Editorial Office:** Lines 40-41: specify which target gene has been used for the identification of Acanthamoeba isolate, see for example lines 64 and 172.

**Response to Editor Office:** As Editorial Office suggested, see example lines 64-172, 18S rRNA target gene has been used for the identification of *Acanthamoeba* isolate.

**Editorial Office:** Line 78: delete “in water samples.”

**Response to Editor Office:** As Editorial Office suggested, in line 78 “in water samples” was deleted.

**Editorial Office:** Lines 277-278 and 281-282: I suggest deleting what concerns on genotypes, and possibly including this information in the specific paragraph on this topic.

**Response to Editor Office:** As Editorial Office suggested, in lines 277-278 and 281-282 was deleted what concerns on genotypes.

**Editorial Office:** Line 466: delete "a human pathogen"

**Response to Editor Office:** As Editorial Office suggested, in line 466 “a human pathogen” was deleted.

Finally, we are sincerely grateful to both reviewers and editors for your time and interest in reviewing and accepting to publish our manuscript. We look forward to having our manuscript accepted and published with Plos one.
